# Botanical Report

**Published:** 1811-12

**Authors:** 


### BOTANICAL REPORT.

We are glad to accept, at irregular periods, now and then, a Number of the BOTANIST'S REPOSITORY. Since we last mentioned this work in June, we have received only one number, and that one less interesting than some others. We shall proceed to enumerate its contents.

Trichilia *odorata*. Native of the West Indies, and consequently with us an inhabitant of the bark-stove. It corresponds so well with Swartz's character of *moschata*, that we suspect it to be the same; for though described here, as having four petals, it appears by the figure to be monopetalous. Sloane's figure referred to by Swartz as a synonym of his *moschata* will not decide the question. Perhaps though said by our author to be a West-Indian plant, it may after all be a native of New Holland.

DAVIESIA *latifolia*. An elegant shrub of the papilionaceous order, nearly related to D. *corymbosa* of Dr. Smith. The fine golden flowers are produced in long upright racemes from the axils of the leaves. Native of New Holland; and communicated by Mr. Milne from Fonthill; but is likewise to be met with in some of the nurseries about town.

Carex *Fraseri*. We have mentioned this singular plant before in our Report of the Botanical Magazine. In the representation given here both edges of the leaf are equally crenulate, and not quite entire on the inner margin, as described and figured in the Botanical Magazine.

Heliconia *Bihai*, or Wild Plantain-tree. There is a very good figure of this plant in Thomson's Botany displayed. The younger Linnæus mistook one of the species of Strelitzia for this plant, and his alterations of the specific character consequently belong to that. But although Swartz in his *Observationes* had long ago pointed out this error, yet in the latest publication we have of a general system of vegetables, viz. *Persoon's Synopsis*, the corolla is said to be *crocea*, the the nectarium *cæruleum*; characters belonging to Strelitzia *Regeniæ* and not at all to this plant, so much easier it is to mislead than to set right again.

We have yet two numbers of the Botanical Magazine unnoticed in our Report; the contents of which are

Lilium *monadelphum*. A new species of Lily, of the same colour and form nearly as the yellow variety of Lilium *Pomponium*. Native of Mount Caucasus.

Watsonia *strictiflora*. A new species i oduced from the Cape by the Hon. Wm. Herbert.

Moræa *Sisyrinchium*. This bulbous-rooted flower is a native of the southern parts of Europe and the northern of Africa; and was well known in our gardens in the time of Parkinson and Gerard, yet of late years it seems to have been quite lost. The present drawing was made from a plant received from Gibraltar by Mr. Vere of Kensington Gore. The older botanists saw the difference in this plant and Iris, and called it Sisyrinchium. Linnæus and most botanists since his time have considered it as a species of the Iris. And Mr. Ker, in the Annals of Botany, first added it to Moræa; in which he has been followed by the author of the new edition of Hortus Kewensis. Before the labours of Mr. Ker indeed the distinction between Moræa and Iris were not at all understood; and the only solid and certain character which distinguishes them this author himself now places in the bulbo-tuberous root of Moræa; for want of which Moræa *Iridoides* is now directed to be added to Iris with the specific name of *Moræoides*. It must be acknowledged that this is departing from the Linnæan principle of taking the generic character from the parts of fructification only.

Allium *obliquum*; a very rare species of Garlic, which Mr. Ker has not observed in any collection but that of Mr. Haworth. In a note subjoined to this article, Mr. Ker has referred the plant figured in the Rare Plants of Hungary under the name of Allium *ampeloprasum*, and which he had before considered as variety $\beta$ of that species, to Allium *arenarium*, of which latter species he is now convinced that it is a mere variety without bulbs, and a fresh proof of the fallacy of distinguishing the species of this genus by their having bulbiferous or capsuliferous umbels. We are however of opinion, from long observation, that, as cultivated in our gardens, the capsuliferous and bulbiferous species continue very constant to their character. Mr. Ker may nevertheless be very right in his opinion, because however constant the character may remain in the same climate, it does not follow that the whole may not depend upon climate: and the capsuliferous species in the south may become bulbiferous in the north, and vice versa, the same species that are bulbiferous in a northern may be capsuliferous in a southern climate.

Bryophyllum *calycinum*. A genus first constituted by Mr. Salisbury in the Paradisus Londinensis. It received its name from the very curious circumstance, that it puts forth a germinating bulb from each crenature of the leaf. Thus, in attempting to dry this plant by placing it between folds of paper. Dr. Sims found that little bulbs were produced from each crenature, though there was no appearance of them before. Differs from cotyledon in being octandrous, and having the limb of the corolla divided into four instead of six segments, from Calanchoe in having the filaments placed in one equal row. This figure is beautifully drawn, engraved, and coloured, and appears to us to equal the expensive figures of the Hortus Schoenbruensis.

Gentiana *septemfida*. A mere variety of the one figured before in the same work, and apparently repeated here by an oversight.

Liatris *spicata*. Native of North America, whence it was introduced by Mr. John Fraser. Serratula *spicata*, given as a synonym of this, has however been in our gardens long, and appears to us to be a taller plant, with darker-coloured flowers.

Carolinea *minor*. Probably the first plant of this genus that has ever flowered in this country. Introduced by Dr. Anderson from Guiana, and brought to flower by Messrs. Loddiges of Hackney.

Schisandra *coccinea*. *Sanguinea* or *miniata* would have been better; but the name was given by Michaux, who first described and figured this plant in his Flora Boreali-Americana. This very rare and singular plant was communicated by John Walker, esq. of Arno's Grove, Southgate. It is a monoicous plant, but unfortunately produced only male flowers.

Gentiana *macrophylla*; nearly akin to G. *crueiata*. Dr. Sims has called this plant by the English name of *long-leaved*. We observe, with some surprise, that in the new edition of the Hortus Kewensis it is called *broad-leaved;* though the leaves are long and narrow, and by no means deserving the epithet of broad, nor was *macros* commonly used in composition by the Greeks in any other sense than to denote length.

Aloë *serrulata* of Haworth. For our own part, though we have no objection to having a good number of figures for our money, we should have been quite as well pleased, had this been made into a double plate, when it might have had the advantage of a miniature outline of the whole plant, of which we have repeatedly expressed our decided approbation.

Pitcairnia *bracleata β. sulphurea*. Professor Swartz, in his *Prodromus*, characterised this genus under the name of Hepetis. And L'Heretier, in his *Sertum Anglicum*, dedicated it to the honour of Dr. William Pitcairn. Both these publications were printed in 1788. The latter name has been pretty generally adopted; but Schreber, in his edition of the *Genera plantarum*, has retained that of Hepetis. We may make the same observation upon this as the latter; a miniature outline of the whole plant, though it doubled the cost, would have exceeded in value in a still greater proportion.

Aloë *arachnoides δ. translucens*. Haworth considers this a distinct species, in which he has been followed in the Hortus Kewensis. Mr. Ker makes it only a variety. Our opinion is, that while plants so distinct in external habit as the different species of aloe, are included under under one genus, it seems most natural to consider such as so nearly resemble one another, as varieties; but where the divisions of this genus, which we hinted at in a former Report, separated into so many distinct genera, all the four varieties, as they are called, of *arachnoides*, would be by general consent considered as so many species, as there can be little reason to suppose that they are really seminal varieties from the same stock.

Aletris *farinosa*. This plant, a native of Virginia, is the one on which Linnæus first founded his genus Aletris, he afterwards added several species from the Cape, which have been since separated under the names of Veltheimia and Tritoma. The whole genus is now limited to the species here figured, another from the same country, and a third from Japan.

This number finishes the 34th volume of this extensive work, containing 1418 figures of plants, all drawn and coloured from nature, equalling in accuracy, and often in elegance, the most expensive botanical figures.

